# Polymethylmetacrylate Cement Augmentation of the Coccyx (Coccygeoplasty) for Fracture: A Case Report

**DOI:** 10.4274/balkanmedj.galenos.2020.2020.4.68

**Published:** 2020-10-23

**Authors:** Ezgi Akar, Orkun Koban, Ahmet Öğrenci, Mesut Yılmaz, Sedat Dalbayrak

**Affiliations:** 1 Clinic of Neurosurgery, Haydarpaşa Numune Training and Research Hospital, İstanbul, Turkey; 2 Department of Neurosurgery, Okan University School of Medicine, İstanbul, Turkey; 3 Department of Neurosurgery, Neurospinal Academia, İstanbul, Turkey

**Keywords:** Coccydynia, coccygeoplasty, fracture, osteoporosis

## Abstract

**Background:**

Coccydynia is a painful condition of the sacrococcygeal region, with symptoms associated with sitting and rising from a seated position. It is frequently related to trauma and idiopathic causes, and the pain is mostly chronic. Percutaneous vertebroplasty and sacroplasty are the methods that are widely used for treating compression fractures and sacral insufficiency fractures, respectively. However, the success of polymethylmethacrylate injection in the treatment of osteoporotic coccyx fractures and coccydynia is still unknown.

**Case Report:**

A 68-year-old man was admitted to our clinic with complaints of pain in the sacrococcygeal and perianal regions. In the imaging studies, a fracture line in the fifth sacral and first coccygeal segments was observed as evidenced by a bony edema. Since the patient’s pain did not improve with conservative methods, we treated him with coccygeoplasty. No complication was encountered. The day after the operation, he was discharged from the hospital with complete pain relief. The patient confirmed having no pain on the third postoperative month and so did not need any analgesics.

**Conclusion:**

Coccyceoplasty may be a good treatment option for retractable pain in patients with acute or subacute osteoporotic coccygeal fractures and coccydinia with edema.

There are several possible causes associated with the tailbone pain, but most of the reasons are idiopathic. The most frequently observed causes are trauma, repetitive micro trauma, obesity, and rapid weight loss (1). Coccydynia, or pain in the region of the coccyx, occurs five times more frequently in women than in men. This is probably due to the higher body mass index in women (2). Coccydynia is either associated with coccyx fractures or with posterior sacrococcygeal subluxation from repeated or prolonged sitting loads and chronic traumas (3). Percutaneous vertebroplasty is a well-known method used in treating vertebral body compression fractures and is also effective in immediate pain control. Besides, sacroplasty is a minimally invasive procedure for treating pathological fractures of the sacral vertebral body. The goal of sacroplasty is to relieve pain and stabilize fractures (4). Treatment of osteoporotic coccyx fractures by polymethylmethacrylate (PMMA) injection is known as coccygeoplasty and has been reported in the literature only once (5). Coccydynia is not a rare condition, and lots of patients may benefit from PMMA augmentation (i.e., coccygeoplasy). To the best of our knowledge, this is the second report of coccygeoplasy, which aims to contribute the knowledge of indications, technique, and outcomes of this procedure.

## CASE REPORT

**Figure 1:**
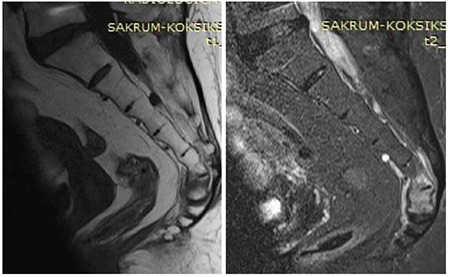
Preoperative images (sagittal T1 and contrast-enhanced magnetic resonance imaging) show coccygeal fracture and bone edema.

**Figure 2:**
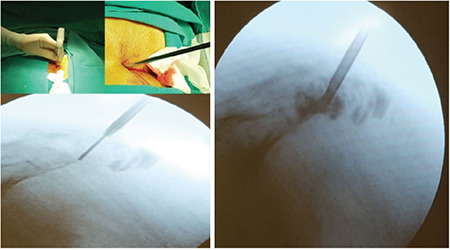
Perioperative fluoroscopy images.

**Figure 3:**
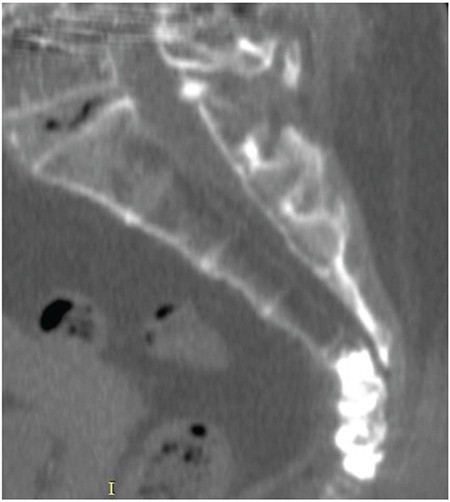
Computed tomography images after coccygeoplasty.

A 68-year-old man was reported to our clinic with complaints of chronic pain in the sacrococcygeal and perianal regions. His pain was aggravated by sitting or during defecation. The pain would stop while changing his position, from sitting to standing, or when he was lying. Although there was no history of major trauma, the patient reported occasional slipping and falling. He was hypertensive and suffered from type 2 diabetes mellitus. He had L3-L4-L5 decompression and stabilization due to lumbar spinal stenosis. Imaging revealed osteopenic bone structure. Upon physical examination, the sacrococcygeal region particularly was sensitive to direct touch. His neurological examination was normal. In the imaging studies [lumbosacral magnetic resonance imaging (MRI) and X-ray], a fracture in the fifth sacral and first coccygeal segments was observed as evidenced by a bony edema and coccyx angulation (Figure 1). The patient was first treated with non-steroidal anti-inflammatory drugs (NSAIDs), and donut pillows were recommended. During the follow-up period, two weeks later, pericoccygeal local anesthetic and steroid injections were administered to the patient, but it did not relieve the pain. The pain was severe enough to affect his daily life. As the conservative treatment did not control the pain, the patient was evaluated for invasive procedures. After informing the patient about the benefits and possible risks of the procedure, the decision was taken to perform coccygeoplasty using PMMA cement. The patient was placed on the operation table in the prone position, administered with spinal anesthesia, and sedated. His pelvis and abdomen were supported with rolls and pads. By placing C-arm fluoroscopy over the operation table, the entry point was determined at the midline using anteroposterior and lateral planes (which corresponded to the painful area of the sacrococcygeal junction) targeting to fill the first and second segments of the coccyx with an angle of about 45 degrees using an 18-gauge Chiba needle. Cannulas were placed at the entry points by checking on the lateral and anteroposterior projections. The projections were checked by fluoroscopy, and approximately 2.5-3 mL of PMMA cement injection was administered (Figure 2). After carrying out the final fluoroscopic controls in the lateral and anteroposterior projections, the operation was completed. No complication was encountered (Figure 3). Postoperatively, the patient was mobilized on the same day. On the first day after the operation, he was discharged from the hospital with complete pain relief. The patient confirmed having no pain on the third postoperative month and so did not need any analgesics.

The ethics committee of our hospital did not require written approval, as there was no use regarding patient identity.

## DISCUSSION

Coccydynia is defined as a pain in the coccygeal region, and the pain is near the anal region and between the buttocks. It is also called tailbone pain. Although its real incidence is unknown, it accounts for less than 1% of all lumbosacral region pains (6). Obesity, female gender, rapid weight loss and internal (e.g., childbirth) and external (e.g., falling backward) traumas, minor traumas (e.g., sitting on a hard and inconvenient surface), nontraumatic causes (anomalies in the mobility of the sacrococcygeal joint, degenerative phenomena, infectious causes, etc) can be the related factors (6).

The pain was aggravated by sitting, standing for a long time, and defecation. Tenderness was observed at the coccyx during physical examination. During imaging studies (X-ray, computed tomography, and MRI), fracture lines and/or the sacrococcygeal joint’s hyper/hypomobility, angulation, and the bone [especially in Short tau inversion recovery (STIR) sequence on MRI] and surrounding soft tissue in edema can be observed (7). The cause of coccydynia in our case was probably micro traumas due to falling, and bone edema in STIR MRI suggested subacute fracture. In coccydynia, X-ray is recommended on sitting and standing positions to evaluate the instability of the sacrococcygeal joint. However, in several centers, it is evaluated via X-ray in the standing position; as such the diagnosis and cause of coccydynia can be missed (8). We recommend X-ray in both sitting and standing positions for patients with pain in the coccygeal region.

Whatever maybe the cause of coccydynia, it can be kept under control by conservative methods such as physical therapy, manipulation of coccyx, and analgesic treatments (NSAIDs, acetaminophen, opioids, etc), U wedge or doughnut-shaped coccygeal cushions (7,8). However, in some cases of coccydynia, which are less frequently observed like our present osteoporotic coccygeal fracture case, conservative methods are not sufficient (about 10% of the cases) (5,6). Interventional methods can be applied in patients whose pain does not regress with conservative methods. These methods are caudal epidural steroid injections, recurrent pericoccygeal local anesthetics and/or steroid injections, ganglionic parasympathetic nerve blocks, and selective radiofrequency ablation of the coccygeal nerve procedures (8,9). It was reported that pain control was achieved in 59% of the cases only by using injections; the success rate was stated to be 85% when injection and manipulation were combined. However, there is a relapse rate of about 28% (10). Surgical procedures must be considered in cases when there is no response received from all other treatment methods. Surgical treatment of coccydynia is known as coccygectomy, which is the amputation of the coccyx just proximal to the sacrococcygeal junction or below S5 (6,8).

Coccygectomy can be performed in a limited number of cases if other treatments fail. This surgical procedure has several risks such as postoperative scar infection, osteomyelitis, persistent pain, and pelvic floor prolapse (4,6,8). Acute or subacute osteoporotic fractures or edematous coccyx that appears in MRI can be considered as cases suitable for coccygeoplasty. Dean et al. (5) defines coccygeoplasty as the treatment of applying cement via percutaneous vertebroplasty in a case with persistent pain due to osteoporotic coccygeal fracture. They considered that conservative methods remained insufficient in treating coccydynia due to osteoporotic fractures and recommended coccygeoplasty method to control pain in such cases (5). This method is similar to vertebroplasty and sacroplasty and ensures pain control as well as mechanical stabilization of fracture by applying PMMA cement. This technique can be rapidly and safely applied to a patient with osteoporotic coccygeal fracture who has refractory pain (5,10). The neurological complication risk of this procedure is very low since there is no spinal canal at the coccygeal level. This procedure can cause painful defecation complications if cement leaks to the pericoccygeal region. In order to prevent this, an operation is recommended to be carried out by using fluoroscopy imaging at a lateral and anteroposterior plane (5,7,8). Coccygeoplasty was performed with the induction of spinal anesthesia to our patient with osteoporotic coccygeal fracture. In the method described by Dean et al. (5), coccygeoplasty was carried out with two separate cannulas set at the two coccyx segments from the right and left. In our case, we have carried out the same procedure from a single midline entrance. Obtaining the same result from a single entrance made the procedure less invasive for the patient. Although there are many alternatives for the treatment of coccydynia, coccygeoplasty is the recommended choice of treatment for osteoporotic coccygeal fractures and coccydynia patients with edema seen in STIR MRI. There are many advantages of coccygeoplasty: It is a successful method for pain control; early mobilization can be achieved through this procedure; there is less morbidity; and the length of hospital stay is short. When general anesthesia is not administered, the risks due to anesthesia can be excluded. Our patient was observed to be pain-free in a three-month follow-up, and the long-term result of coccygeoplasty was found to be successful.

There is still not a single method that can be considered as the standard in treating coccydynia. It is usually controlled by conservative measures such as physical therapy, manipulation of the coccyx, analgesic treatments, and coccygeal cushions. However, these methods may be insufficient in osteoporotic coccygeal fractures. Coccygeoplasty is recommended as a successful method for the treatment of retractable pain in the treatment of acute or subacute osteoporotic coccygeal fractures and coccydynia with edema.
